# Critical care hepatology: definitions, incidence, prognosis and role of liver failure in critically ill patients

**DOI:** 10.1186/s13054-022-04163-1

**Published:** 2022-09-26

**Authors:** Aritz Perez Ruiz de Garibay, Andreas Kortgen, Julia Leonhardt, Alexander Zipprich, Michael Bauer

**Affiliations:** 1 University of Strasbourg, CNRS, Immunopathology and Therapeutic Chemistry, UPR 3572, 67000 Strasbourg, France; 2 Department of Anesthesiology and Intensive Care Medicine, Jena University Hospital, Am Klinikum 1, 07747 Jena, Germany; 3 Department of Internal Medicine IV (Gastroenterology, Hepatology, Infectious Diseases), Jena University Hospital, Jena, Germany; 4 Present Address: ADVITOS GmbH, Munich, Germany

**Keywords:** Acute liver failure, Acute-on-chronic liver failure, Secondary liver failure, Intensive care unit, Multiple organ failure

## Abstract

Organ dysfunction or overt failure is a commonplace event in the critically ill affecting up to 70% of patients during their stay in the ICU. The outcome depends on the resolution of impaired organ function, while a domino-like deterioration of organs other than the primarily affected ones paves the way for increased mortality. “Acute Liver Failure” was defined in the 1970s as a rare and potentially reversible severe liver injury in the absence of prior liver disease with hepatic encephalopathy occurring within 8 weeks. Dysfunction of the liver in general reflects a critical event in “Multiple Organ Dysfunction Syndrome” due to immunologic, regulatory and metabolic functions of liver parenchymal and non-parenchymal cells. Dysregulation of the inflammatory response, persistent microcirculatory (hypoxic) impairment or drug-induced liver injury are leading problems that result in “secondary liver failure,” i.e., acquired liver injury without underlying liver disease or deterioration of preexisting (chronic) liver disease (“Acute-on-Chronic Liver Failure”). Conventional laboratory markers, such as transaminases or bilirubin, are limited to provide insight into the complex facets of metabolic and immunologic liver dysfunction. Furthermore, inhomogeneous definitions of these entities lead to widely ranging estimates of incidence. In the present work, we review the different definitions to improve the understanding of liver dysfunction as a perpetrator (and therapeutic target) of multiple organ dysfunction syndrome in critical care.

**Graphic Abstract:**

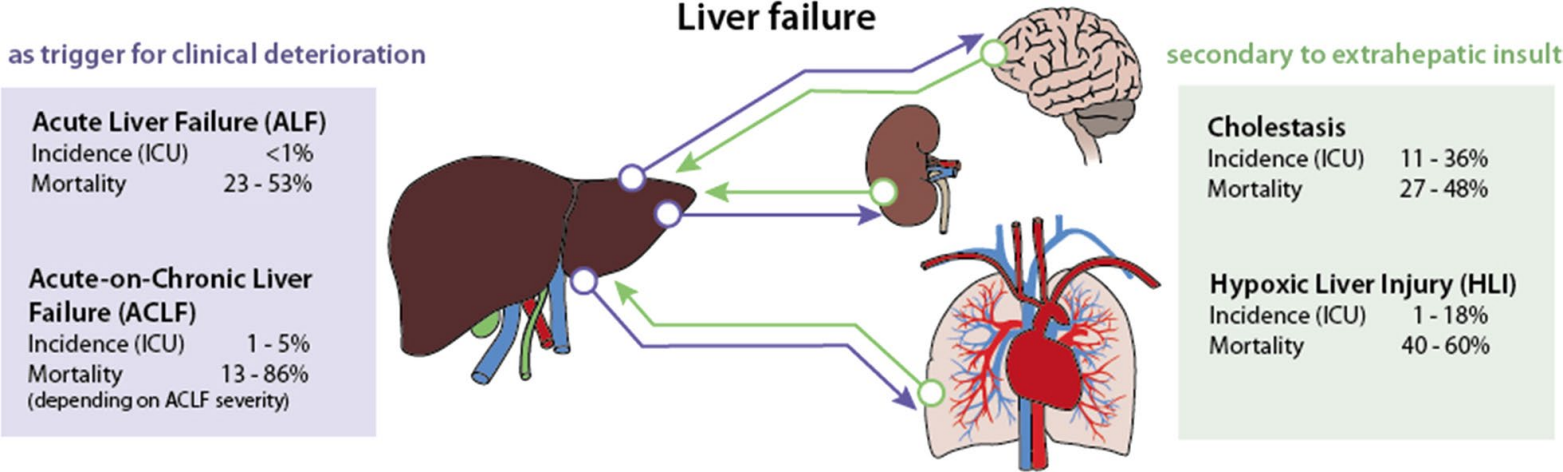

## Introduction

The definition of liver dysfunction is far from a global consensus. Typically, liver failure is divided into two major entities depending on the presence or absence of preexisting liver disease. Acute liver failure (ALF) is rare, occurs in the absence of previous liver damage, has a clear etiology and is classified according to the interval between the appearance of jaundice and the onset of hepatic encephalopathy into acute, subacute and hyperacute processes [1–3]. ALF is managed according to guidelines, which account for transplant needs [1, 4]. In contrast, acute-on-chronic liver failure (ACLF) is triggered by acute hepatic decompensation in patients with preexisting chronic disease [5]. ACLF definitions vary depending on the issuing consortium [6–8]. Apart from ALF and ACLF, secondary liver injury without underlying liver disease in response to hypoxic, toxic or inflammatory insults represents the most common form of hepatic dysfunction in the intensive care unit (ICU), and it commonly manifests as cholestasis and hypoxic liver injury [9, 10].

In ALF, the liver is triggering clinical deterioration, i.e., extrahepatic organ dysfunction develops due to impaired liver function. The situation in ACLF is more complex: A previous chronic liver disease worsens to liver failure by primary hepatic deterioration [alcoholic hepatitis, viral hepatitis, drug-induced liver injury (DILI)] or by secondary hepatic deterioration due to extrahepatic events (for example, sepsis). Whether alterations are classified as “dysfunction,” “injury” or “failure” depends on the surrogate marker and the score used. For instance, when applying bilirubin as a common marker, the definition of acute liver injury (ALI) by Koch et al. uses a value higher than 3 mg/dl (51.3 µmol/l) to define injury [11]. The Sequential Organ Failure Assessment (SOFA) Score divides dysfunction and failure using thresholds of 1.2 mg/dl (20.5 µmol/l) and 6.0 mg/dl (102.6 µmol/l) [12], respectively. Levels above 2 mg/dl are otherwise frequently used as pragmatic cutoffs to assess jaundice and cholestasis [6, 9].

Experimental data on immunologic, regulatory and metabolic functions of the liver suggest a role of the liver as a “perpetrator” rather than a “victim” of “host response failure” and multiple organ failure [13–15]. In any case, liver dysfunction and failure are clearly of utmost importance in the ICU as they affect at least 20% of patients and significantly increase the risk of death [16, 17].

The following overview will discuss ALF, ACLF and secondary liver failure. A thorough review and description of the pathophysiology and management can be found elsewhere [3, 9, 18–20].

## Acute liver failure (ALF)

### Definition

Acute liver failure was first defined in 1970 to describe a rare and potentially reversible critical illness caused by “severe liver injury in the absence of prior liver disease with hepatic encephalopathy occurring within 8 weeks from the appearance of first symptoms” [21]. In general, the duration of the symptoms should not be longer than 26 weeks to be considered ALF [22].

Since the first definition of ALF, several authors have classified ALF according to the interval between the appearance of jaundice and the onset of hepatic encephalopathy (HE) (Table 1), e.g., defining “fulminant” for appearance of hepatic encephalopathy in the first 2 weeks and “subfulminant” when occurring until week 12 [23]. O´Grady employed the terms “hyperacute,” “acute” and “subacute” for an onset of hepatic encephalopathy within 1, 4 or between 5 and 12 weeks, respectively [2]. The Japanese consensus classifies ALF into “acute liver failure without hepatic coma” (HE < Grade II) and “acute liver failure with hepatic coma” (HE ≥ Grade II). The latter distinguishes between the “acute type” and “subacute type,” with hepatic encephalopathy developing within 10 days or between 11 and 56 days, respectively [24]. If hepatic encephalopathy occurs later, the disease is known as late-onset hepatic failure (LOHF) [4]. All these classifications can even be further complicated if the etiology of ALF [22, 25] is taken into consideration, which can lead to lack of comparability of clinical trials [26].

**Table 1:** Definition, incidence, mortality and prognostic clinical features cutoff for mortality increase in the different types of liver injury

Liver failure	Defined by	Subtypes/classification	Incidence (% ICU patients)	Mortality (28 and 90 day)	Prognostic clinical features	Cutoff for mortality increase
ALF	Hepatic Encephalopathy Coagulopathy: INR > 1.5 Absence of previous liver injury Duration < 26 weeks	According to the interval from jaundice to HE appearance: Bernuau: Fulminant: < 2 weeks Subfulminant: 2–12 weeks O´Grady: Hyperacute: < 1 week Acute: 1–4 weeks Subacute: 5–12 weeks Japanese consensus (if HE < II: without hepatic coma; If HE ≥ grade II: with hepatic coma): Fulminant: 0–8 weeks Acute: 0–10 days Subacute: 11–56 days LOHF: > 56 days	< 1%	Up to 50%	Grade of HE	Acute/hyperacute versus subacute/LOHF
					Extrahepatic organ failure	AKI
					Age	< 10 or > 40 years
					Lactate	≥ 4 mmol/l
					Bilirubin (non-paracetamol)	> 17 mg/dl
					Arterial ammonia	> 100 µmol/l
ACLF	EASL: Acute deterioration: Usually related to a precipitating event From extrahepatic origin or Secondary to superimposed liver injury Preexisting liver disease: Chronic liver disease High 90-day mortality due to multisystem organ failure	According to the presence of extrahepatic failure: ACLF 1: Single kidney failure, or Single liver/coagulation/circulatory/respiratory failure and SCr: 1.5–1.9 mg/dl or Mild-to-moderate HE, or Single cerebral failure and SCr 1.5 mg/dl ACLF 2: 2 organ failures ACLF 3: ≥ 3 organ failures	1–5% (24–40% of patients with cirrhosis admitted to hospital)	28-day: 34% ACLF 1: 22% ACLF 2: 32% ACLF 3: 77% 90-day: 51% ACLF 1: 41% ACLF 2: 52% ACLF 3: 79%	Bilirubin	6–12 mg/dl
					HE	Grade I-II
					INR	2.0–2.5
					MAP	< 70 mmHg
					Creatinine	2.0 mg/dl in single kidney failure, or 1.5–1.9 mg/dl in single non-kidney organ failure
					Age	
					WBC count	Infection [6]
					Number of organ failures	≥ 2
					Respiratory function	PaO_2_/FiO_2_: 200–300, or SpO_2_/FiO_2_: 214–357
	NACSELD: Cirrhosis and two extrahepatic organ failures Organ failures are defined as (1) Shock (2) Grade III/IV hepatic encephalopathy (HE) (3) Need for dialysis (4) Mechanical ventilation	N.a	N.a	According to the number of organ failure and infection 1 OF: 10%/20% 2 OF: 16%/38% 3 OF: 35%/58% ≥ 4 OF: 0%/76%	Number of organ failure	≥ 2
	APASL: Acute hepatic insult: Jaundice: bilirubin ≥ 5 mg/dL Coagulopathy: INR ≥ 1.5 or PT activity < 40% Complicated within 4 weeks by Ascites and/or HE Preexisting liver failure: Diagnosed or undiagnosed Chronic liver disease/cirrhosis High 28-day mortality Organ failure other than liver is not part of the definition	According to AARC Score [47], which defines the grade of liver failure: Grade I (mild): 5–7 Grade II (severe): 8–10 Grade III (very severe): 11–15	1–5% (24–40% of patients with cirrhosis admitted to hospital)	28-day: 33–44% Grade I: 12.7% Grade II: 44.5% Grade III: 85.9% 90 day: 47–53%	HE	Grade III-IV
					Infection	
					INR	1.8–2.5
					Lactate	1.5–2.5 mmol/l
					Creatinine	1.1–1.5 mg/dl or AKIN Stage 1
					Age	
					WBC count	
					Obesity	
					“Golden window”	Sepsis, MOF
Secondary Acquired Liver Injury	Cholestasis: Altered bile excretion, synthesis or secretion Bilirubin > 2 mg/dl (no consensus exist)	According to the mechanism: Extrahepatic Intrahepatic	11–36%	27–48%	Bilirubin Bile acids Concomitant syndromes	> 2 mg/dl ≥ 5.2 µmol/l increase Sepsis
	Hypoxic Liver Injury: Respiratory, cardiogenic or circulatory shock Elevation of transaminases > 20-fold from the reference value Absence of underlying liver injury	According to precipitating event: Sepsis Cardiogenic shock Parenteral nutrition	10%	40–60%	SOFA score: INR Peak arterial ammonia ICG-PDR Concomitant syndromes	≥ 11 > 2 > 75 µmol/l < 9%/min Sepsis

*ALF* acute liver failure, *ACLF* acute-on-chronic liver failure, *EASL* European Association for the Study of the Liver, *APASL* Asian Pacific Association for the Study of the Liver, *LOHF* late-onset hepatic failure, *SCr* serum creatinine, *AKIN* acute kidney injury network, *INR* international normalized ratio, *MAP* mean arterial pressure, *ICU* intensive care unit, *HE* hepatic encephalopathy, *AKI* acute kidney injury, *MOF* multiple organ failure, *ICG-PDR* indocyanine green plasma disappearance rate and *WBC* white blood cell

### Incidence and mortality

Estimates based on data from transplant units from the European Union suggest that 8% of liver transplants (LTx) are due to ALF, either caused by viral infection (19%), DILI (18%), toxic insults (4%), traumatic events (3%) or unknown causes (56%) [27]. However, a trend toward an increase in DILI and a decrease in viral etiologies has been documented [1, 28, 29]. Overall, ALF occurs in less than 10 cases per million inhabitants per year [3]. In-hospital mortality remains high with rates between 23 and 53% [9].

### Prognostic clinical features

Wlodzimirow et al. identified and characterized different prognostic models for mortality in ALF patients [30]. The variables more commonly included in the final model of the studies investigated were hepatic encephalopathy (45%), prothrombin time (45%), bilirubin (40%), age (40%) and creatinine (25%) [30]. A recent study revealed that M30, a cleavage product of cytokeratin-18 caspase, most accurately identified patients who would require LTx or die [31].

- *Hepatic encephalopathy* HE remains the essential clinical hallmark, and its presence, even at low grade, is indicative of poor prognosis [1]. HE is primarily a clinical diagnosis. The joint EASL/AASLD guidelines suggest that if ammonia levels are normal, the diagnosis of HE is in question [32]. EEG provides information on the severity of HE in both cooperative and especially in uncooperative patients but is nonspecific [33]. Arterial ammonia concentration in whole blood on admission to the ICU is an independent risk factor for both encephalopathy and intracranial hypertension [34].
- *Creatinine* The presence of extrahepatic organ failure, especially acute kidney injury, has been shown to increase mortality rates [3, 35, 36]. The occurrence of concomitant kidney failure (i.e., multiple organ failure) urges admission to the ICU [1].
- *Severity of liver injury (reflected in prothrombin time or bilirubin)* Changes in coagulation factors, such as INR, reflecting injury to the hepatocellular synthesis machinery, are of prognostic value. Similarly, serum bilirubin, reflecting injury to the excretory machinery of the hepatocyte, serves as a prognostic marker in ALF of non-paracetamol etiology, but has no value in paracetamol-induced ALF or even other causes of hyperacute liver failure due to the time required for bilirubin levels to build up [37].
- *Other factors* These may include age (e.g., < 10 or > 40 years) and lactate levels (e.g., > 4 mmol/l), as indicated in the King’s College Criteria or the recent guidelines in the UK for the assessment of the need for LTx [2, 38]. Lactate, together with bilirubin and etiology, is part of the BiLE score, which has recently shown a good predictive value in a cohort of 102 ALF patients [37]. Other scoring systems are available for rare diseases, for example, the TIPS-BSC prognostic index [39].

## Acute-on-chronic liver failure (ACLF)

### Definition

ACLF is a syndrome affecting multiple organs, including new worsening of liver function, defined by an acute hepatic decompensation in patients with preexisting chronic liver disease [5]. Even if the definition provided by the European Association for the Study of the Liver (EASL) and the International Chronic Liver Failure (CLIF) Consortium is the most employed [6], there is no single agreed definition for ACLF. Since the first report in 1995 [40], different attempts have been made in Europe [6, 41], Asia [7, 42, 43] and North America [8]. A consensus definition has been proposed, but has not yet been clinically validated [5]. Table 1 summarizes the current definitions of ACLF. A recent paper even highlighted three different stages in acutely decompensated cirrhosis, i.e., stable decompensated cirrhosis, unstable decompensated cirrhosis and ACLF [44]. In this study, ACLF was differentiated from decompensated cirrhosis by the presence of organ failure and systemic inflammation.

According to an investigation of 1343 patients with decompensated cirrhosis (CANONIC study), the EASL included the presence of at least renal dysfunction (creatinine > 1.5 mg/dl) or hepatic encephalopathy (grade I or II), together with a high risk of death due to multiple organ failure, as criteria to define each of the subgroups of ACLF [6]. These organ failures were first defined according to the SOFA score (Table 2). Depending on the number of failing organs, different subgroups were established (Table 1).

**Table 2:** Summary of scores employed in the ICU to characterize hepatic and extrahepatic organ failures

Score	Use	Range	Definition of organ failure						Predicted mortality	References
			Liver	Kidney	Coagulation	Respiratory	Circulatory	CNS		
APACHE-II	Assess the baseline risk groups being compared in clinical trials and determine prognosis on all patients newly admitted to the ICU	0–77	Cirrhosis	Need for dialysis	–	severe exercise restriction or respiratory dependency	NYHA Class IV	Based on GCS	10: 15% 20: 40% > 34: 85%	[121]
	Components:	History of severe organ failure (Heart Failure Class IV; cirrhosis; chronic lung disease, or dialysis-dependent); Age; Temperature; MAP; pH; Heart rate/pulse; Respiratory rate; Sodium: Potassium; Creatinine; Acute renal failure; Hematocrit; White blood cell count; GCS; and FiO_2_								
SOFA	Determine level of organ dysfunction and mortality risk in ICU patients	0–24	Bilirubin ≥ 6 mg/dl	Creatinine ≥ 3.5 mg/dl	Plat < 50,000	PaO_2_/FiO_2_ < 200 and MV	High-dose vasopressors	GCS < 13	6: 21% 10: 50% > 14: 95%	[12]
	Components:	FiO_2_/PaO_2_ (and MV); Platelets; GCS; Bilirubin; MAP (use of vasopressors); and Creatinine (or urine output)								
CLIF-SOFA	Modified SOFA score, which had been specifically developed for the CANONIC study with patients with cirrhosis hospitalized for an acute decompensation	0–24	Bilirubin ≥ 12 mg/dl	Creatinine ≥ 2.0 mg/dl	INR ≥ 2.5	PaO_2_/FiO_2_ < 200 or SpO_2_/FiO_2_ < 214	Use of dopamine, dobutamine or terlipressin	HE ≥ III	See SOFA Score	[6]
	Components:	FiO_2_/PaO_2_ or SpO_2_/PaO_2_ (and MV); INR; Hepatic Encephalopathy; Bilirubin; MAP (use of vasopressors); and Creatinine (or urine output)								
CLIF-C OF and CLIF-C ACLF	CLIF-C OF: Simpler and validated organ failure score for the diagnosis and grading of ACLF CLIF-C ACLF: Specific prognostic score for ACLF obtained from the combination of CLIF-C OF, age and white blood cell count	6–18	Bilirubin ≥ 12 mg/dl	Creatinine ≥ 2.0 mg/dl	INR ≥ 2.5	PaO_2_/FiO_2_ < 200 or SpO_2_/FiO_2_ < 214	Use of vasopressors	HE ≥ III	ACLF 1: 22% ACLF 2: 32% ACLF 3: 77%	[51]
	Components:	CLIF-C OF: Bilirubin; Creatinine; Need for RRT; HE Grade; INR, MAP (use of vasopressors); FiO_2_/PaO_2_ or SpO_2_/PaO_2_ (and MV) CLIF-C ACLF: Age; White blood cell count; and CLIF-C OF score								
AARC ACLF	Prognostication and timely referral for liver transplantation. The score grades liver failure. The cutoff values for each system failure in this table are based on the definition of the APASL	5–15	Bilirubin ≥ 5 mg/dl	AKIN criteria: Creatinine: increase ≥ 0.3 mg/dL, or ≥ 1.5–2 × from baseline Urine output < 0.5 mL/kg per hour for > 6 h	INR ≥ 1.5	–	–	HE ≥ III	5–7: 12.7% 8–10: 44.5% 11–15: 85.9%	[47]
	Components:	Bilirubin, HE Grade, INR, Lactate, Creatinine								
NACSELD ACLF	Facilitate prognosis determination in both infected and uninfected individuals with cirrhosis		Cirrhosis	Need for RRT	–	Need for MV	Shock: MAP < 60 mmHg	HE ≥ III	1 OF: 37% 2 OF: 49% 3 OF: 64% ≥ 4 OF: 77%	[45]
	Components:	Cirrhosis; Need for RRT; Need for MV; MAP; and HE								
MELD	Determine prognosis and prioritize receipt of liver transplantation	6–40	The MELD score does not define the severity of different organ systems. It is less accurate for mortality prognosis than other scores							[122]
	Components:	Need for dialysis; Creatinine; Bilirubin; and INR								
MELD-Na	The MELD-Na may improve upon the MELD score for liver cirrhosis	6–40	The MELD-Na score does not define the severity of different organ systems. The MELD-Na has been found to have a better fit for mortality prediction compared to the MELD score alone						20: 4% 26: 15% > 32: 65%	[123]
	Components:	Need for dialysis; Creatinine; Bilirubin; INR; and Sodium								
Child–Pugh	Prognosis of patients with cirrhosis	5–15	The Child–Pugh score does not define the severity of different organ systems apart from liver. More recent scores like the MELD score and MELD-Na have become more used given their better prognostic value							[124, 125]
	Components:	Bilirubin; Albumin; INR; Ascites; HE								

*APACHE* Acute Physiology and Chronic Health Evaluation, *SOFA* Sequential Organ Failure Assessment, *CLIF-C* Chronic Liver Failure Consortium, *ACLF* acute-on-chronic liver failure, *OF* organ failure, *AARC* Asian Pacific Association for the Study of the Liver-ACLF Research Consortium, *NACSELD* North American Consortium for the Study of End-Stage Liver Disease, *MELD* model for end-stage liver disease, *HE* hepatic encephalopathy, *Plat* platelet count, *MV* mechanical ventilation, *CNS* central nervous system, *GCS* Glasgow Coma Score, *NHYA* New York Heart Association, *INR* international normalized ratio, *RRT* renal replacement therapy, *MAP* mean arterial pressure

Similar to the EASL description, the North American Consortium for the Study of End-Stage Liver Disease (NACSELD) includes extrahepatic organ failure in its definition [8] (summarized in Table 2). This stratification was validated in a study with more than 500 cirrhotic patients [45].

In contrast, the Asian Pacific Association for the Study of the Liver (APASL) does not include extrahepatic organ failure in its definition, arguing with potential delay in identification of the potential therapeutic window for reversal [7]. In any case, there is no consistent definition of organ failure, as illustrated in Table 2. Moreover, neither patients with an extrahepatic insult as a precipitating factor for ACLF nor patients with prior decompensation are considered in the definition [46].

### Incidence and mortality

Data reported from hospital registries indicate that 24–40% of patients with cirrhosis admitted to the hospital develop ACLF [19]. This accounts for approximately 1–5% of patients admitted to the ICU [9]. Up to 44% and 53% of patients with ACLF died by day 28 and day 90, respectively. In the first 4 weeks, mild ACLF leads to death in 13–22% of patients, whereas moderate and severe failure results in mortality rates of 32–44% and 77–86%, respectively [6, 7, 43, 47]. A recent meta-analysis using the EASL definition to assess the global burden of ACLF demonstrated that 35% of patients with decompensated cirrhosis already presented ACLF at hospital admission, showing a 60% mortality at 90 days [48, 49]. Proven bacterial infection and severe alcoholic hepatitis accounted for almost all (approximately 97%) of acute decompensation and ACLF [50].

### Prognostic clinical features

EASL and APASL use different criteria to predict higher mortality rates. The European Society first used the CLIF-SOFA score, a modified SOFA score for patients with cirrhosis, to define organ “failure” (Table 2). Parameters such as bilirubin, creatinine, HE, INR, PaO_2_/FiO_2_ or S_P_O_2_/FiO_2_ ratios, mean arterial pressure (MAP) and the need for vasopressors were employed to assess damage to liver and extrahepatic organ systems [6]. In the CANONIC study, four groups of patients were defined: the occurrence of ≥ 2 organ failures, the presence of kidney failure alone and the combination of a single non-renal organ failure with kidney dysfunction and/or mild-to-moderate HE [6]. In addition, the impact of the inflammatory response (as reflected in an increased white blood cell (WBC) count) and its early recognition and aggressive management were also recognized to influence the outcome [18]. Afterward, using the same parameters, the score was adapted into the more specific CLIF-C OF score, which was then combined with age and WBC count into the CLIF-C ACLF score, which improved prediction of mortality [51].

The Asian Pacific societies developed their own score based on bilirubin, HE grade, INR, lactate and creatinine (Table 2). This APASL-ACLF Research Consortium (AARC) score was only thought to stratify the degree of liver failure into mild, severe and very severe [47]. The definition of kidney failure follows the acute kidney injury network (AKIN) criteria [52], while no specific reference exists for circulatory or respiratory failures [7]. Prognostic factors such as bilirubin, HE or creatinine are considered, even if different cutoff values are used (Table 2). In the last update, the APASL also included obesity as a risk factor [7].

The approach of NACSELD is even simpler; only the number of organ failures being ≥ 2 is considered the main prognostic factor [8]. In fact, this seems to predict increased mortality better than age, WBC count, serum albumin, MELD score and/or presence of infection [45, 53]. The definition of “failure” does not follow any specific score and is based on the presence of severe HE, low MAP and the need for hemodialysis or mechanical ventilation (Table 2). Hepatic encephalopathy grade and the presence of infection have also been shown to be associated with higher mortality rates [54].

## Secondary acquired liver injury

### Definition

Acquired liver injury without underlying liver disease represents the most common form of hepatic dysfunction in the ICU [9, 10]. It can occur after a hypoxic (e.g., shock), toxic (i.e., hepatotoxic drugs) or inflammatory insult (e.g., sepsis) [55]. There is no common definition for this syndrome, and its diagnosis is mainly based on the elevation of liver parameters, such as transaminases or bilirubin [56].

*Cholestasis* is characterized by altered bile excretion (i.e., extrahepatic cholestasis due to mechanical bile duct obstruction) or impaired conjugation and/or secretion (i.e., intrahepatic cholestasis owing to altered hepatocellular signaling and transport) [9, 55, 57]. This results in an accumulation of bile acids and conjugated bilirubin, together with increased enzymes that indicate cholestasis, such as alkaline phosphatase (AP) or γ-glutamyl transpeptidase (GGT). A consensus on how to define cholestasis in critically ill patients using these routine laboratory parameters does not exist. As defined by the SOFA score [12], a bilirubin threshold > 2 mg/dl is included in the guidelines of the Surviving Sepsis Campaign [58] and has been adopted by many authors [9, 14, 15, 56, 59, 60]. However, elevated bilirubin levels are not specific to cholestasis and might also reflect hemolysis. Lyu et al. [61] showed that 73% of adult cardiac patients supported by veno-arterial ECMO had hyperbilirubinemia (> 3 mg/dl), which may be due to hemolysis in up to 42% of the cases. The combination of AP and GGT with or without bilirubin has been suggested to diagnose cholestasis more specifically [62, 63].

*Hypoxic liver injury* is generally defined based on a clinical setting of circulatory, cardiac or respiratory failure, a substantial increase in transaminases, ranging from > 5 to > 20 times the upper limit of normal (ULN), and the absence of other causes of liver damage [9, 64–70]. It was first reported in 1901 as “central necrosis” [71] and is known as “ischemic hepatitis,” “shock liver” or “hypoxic hepatitis” [67]. The cause is an imbalance of oxygen supply and demand in the liver that results in cell death [64]. More specifically, insufficient hepatic perfusion, including Budd–Chiari perfusion, hypoxemia, poor global oxygen delivery, inadequate oxygen extraction by hepatocytes or an increased metabolic demand, can cause hypoxic liver injury [72–76]. Three subgroups of causes may be distinguished: respiratory failure, cardiac failure and shock/hypotension [1, 67].

### Incidence and mortality

Due to the absence of a consensus definition, heterogeneous epidemiological data for secondary liver injury exist. Cholestasis is present in 11–36% of ICU patients [9], while hypoxic liver injury occurs in 1–18% of cases [69, 70, 77]. For the latter, the incidence increases over 20% in patients with shock [75]. The overall mortality for secondary acquired liver injury is high, ranging between 27 and 48% for ICU patients with cholestasis [56, 76] and between 40 and 60% for hypoxic liver injury [9, 69, 70, 72, 74, 75]. Moreover, in a single-center cohort with 1116 critically ill patients, mortality rates were significantly correlated with the magnitude of transaminases (33.2, 44.4 and 55.4% for peak AST 5–10 × ULN, 10–20 × ULN and > 20 × ULN, respectively) [70].

### Prognostic clinical features

Serum bilirubin is a stable and prevailing marker of liver impairment in the ICU. Its concentration is influenced by bilirubin synthesis, transport, uptake, conjugation and excretion. Ischemic and sepsis-associated cholestasis, drug-induced liver injury and parenteral nutrition are predominant causes of hyperbilirubinemia in the ICU [55]. Bilirubin is a marker of liver dysfunction and a powerful prognostic factor. It has been shown to be linked to infections in surgical patients [78, 79], to an increased mortality among trauma patients [80, 81], sepsis [82] or hematological malignancies [83], to a poor prognosis among ARDS patients [84], or simply to a worse outcome in the ICU [56, 85] (summarized in Table 3).

**Table 3:** Summary of the incidence and the overall mortality associated with increased bilirubin in ICU patients as reported in the literature

Study	Year	Bilirubin cutoff (mg/dl)	Population	Sample size	Incidence %	Mortality %	OR	95% CI		*p*	
Liver dysfunction/cholestasis											
Harbrecht [80]	2002	2	Trauma	2857	7.6	17.0	3.25	1.42–7.45		0.005	
Krammer [56]	2007	2	ICU	38,036	10.9	23.4	1.86	1.71–2.03		< 0.001	
		1–2			19.3	21.3	1.24	1.14–1.34		< 0.001	
		2–3			5.4	26.7	1.494	1.31–1.70		< 0.001	
		3–6			4.3	33.3	2.228	1.94–2.55		< 0.001	
		6–10			1.5	38.5	2.604	2.10–3.23		< 0.001	
		> 10			1.0	46.8	3.991	3.10–5.13		< 0.001	
Jäger [76]	2012	3	Hypoxic liver injury	175	36.0	64.0	2.195**	1.17–4.12		0.014	
Bingold [17]	2015	1.2	ICU	23,795	19.0	N.d	1.335	1.22–1.47		< 0.001	
Dizier [84]	2015	2	ARDS	805	17.6	52.1	1.43	1.28–1.61		< 0.001	
Guo [126]	2015	2*	Intra-abdominal infection	353	41.6	38.8	8.185	3.36–19.94		< 0.001	
Diab [79]	2017	1.2	Infective endocarditis	285	23.9	51.5	5.00	2.48–10.06		< 0.001	
Salojee [81]	2017	2	Trauma	225	21.3	31.3	Not significant				
Pierrakos [117]	2017	1.1–2.0	Infection	8973	16.9	29	1.38	1.18–1.62		< 0.001	
		2.1–6.0			7.8	40	1.71	1.38–2.12		< 0.001	
		> 6			6.5	31	1.54	1.20–1.97		< 0.001	
Han [116]	2021	12–15	Extreme hyperbilirubinemia (≥ 12 mg/dl)	1946	5.7	62.2	Control	Control		Control	
		15–20			5.1	71.7	1.543	0.76–3.14		< 0.001	
		20–30			6.7	81.7	2.714	1.33–5.55		< 0.001	
		≥ 30			0.4	90.7	5.935	2.17–16.20		< 0.001	
Bisbal [83]	2021	2	Hematologic malignancy	893	20.7	45.4	2.26	1.62–3.14		< 0.001	
Juschten [82]	2022	2***	Sepsis	4836	11.6	34.0	1.31	1.06–1.60		0.018	
*Hypoxic liver injury*											
Fuhrmann [77]	2011	> 20-fold TA	ICU	1066	11.1	57.0	4.62	3.63–5.86		< 0.001	
Champigneulle [127]	2016	> 20-fold TA	Out of hospital CA	632	11.4	86.1	4.39	1.71–11.26		< 0.01	
Jung [128]	2017	> 20-fold TA	Cardiogenic shock	172	18.0	68.0	2.52	1.30–4.90		< 0.001	
Iesu [129]	2018	> 20-fold TA	Resuscitated after CA	374	7.2	89.0	16.28****	2.62–81.34		0.003	
Van den broecke [70]	2018	> 5 AST	ICU	1116	1.3	33.2	Not documented				
		> 10 AST			0.9	44.4					
		> 20 AST			1.5	55.4					
Jonsdottir [69]	2022	> tenfold TA	ICU	159	1.6	53%	Not documented				

*OR* odds ratio, *CI* confidence interval, *ICU* intensive care unit, *ARDS* acute respiratory distress syndrome, *N.d* not documented, *TA* transaminases, *AST* aspartate aminotransferase, *CA* cardiac arrest

*Increase of 2 mg/dl from baseline

**OR for complications, not for mortality

***Within the first 2 days after ICU admission

****OR for unfavorable neurological outcome, not for mortality

In the case of cholestasis, total bile acids are also an independent prognostic factor of disease severity [57, 86]. An elevation of 5.2 µmol/l of total bile acids from baseline data demonstrated discriminating value, while mortality was specifically augmented with increases > 10 µmol/l [86]. Horvatits et al. suggested that bile acids could be a better prognostic factor than bilirubin in the ICU. Nevertheless, it remains unclear whether the elevation of bile acids in critically ill patients is a distinct pathophysiological entity or a compensatory mechanism [86].

Regarding hypoxic liver injury, the indocyanine green plasma disappearance rate is an effective tool for assessing liver function. In an observational study with 97 patients, a cutoff value of 9% 48 h after ICU admission demonstrated a significant prognostic accuracy for 28-day mortality [87]. In contrast, bilirubin, due to the later peak time, might not be a reliable marker and is elevated in only one-third of patients with hypoxic liver injury [76]. The indocyanine green plasma disappearance rate outperforms bilirubin as a more sensitive combined indicator of perfusion and excretory liver function in the ICU [88].

Finally, underlying and concomitant syndromes will impact the outcome, such as sepsis or need for organ support [89]. Moreover, the presence of coagulopathy or hepatic encephalopathy has been shown to predict mortality in critically ill patients with hypoxic liver injury [74, 90]. As expected, the severity of organ failure results in higher mortality rates [9, 74].

## Liver injury as a core component of multiple organ failure

The role of the liver in multiple organ failure has been widely studied, especially in sepsis [15]. Due to the extensive number of immunologic, regulatory and metabolic functions, the liver can take the role of the “perpetrator,” triggering an inflammatory response, or that of the “victim” of the host response [13–15].

ALF is rare but frequently results in damage to other organ systems. These include the cardiovascular and respiratory systems, the central nervous system, the kidney, coagulation and the immune system [20]. This also applies to secondary acquired liver injury, since a hypoxic, toxic (e.g., due to commonly used drugs in the ICU [91]) or inflammatory extrahepatic insult triggers hepatic dysfunction.

Each of the organs affected may act either as a precipitating factor or as a target, both paving the way to the worsening of multiple organ failure. This is especially true for the inflammatory response accompanying infection as a prototypical trigger [13, 15, 92].

During ACLF, the release of both damage-associated molecular patterns (DAMPs) linked to inflammation and pathogen-associated molecular patterns (PAMPs) is common [46]. The intensity of inflammation directly correlates with the number of failing organs and outcome [93].

The progressive impairment of circulation is considered as a risk factor for the development of multiple organ failure [94, 95]. The “peripheral arterial vasodilation hypothesis” suggests that during liver failure, portal hypertension and splanchnic vasodilatation emerge [96]. As a result, a reduction in systemic vascular resistance and central hypovolemia arises. Moreover, activation of hormone systems, such as the renin–angiotensin–aldosterone system (RAAS), sympathetic nervous system (SNS) and antidiuretic hormone (ADH), occurs.

**Fig. 1:**
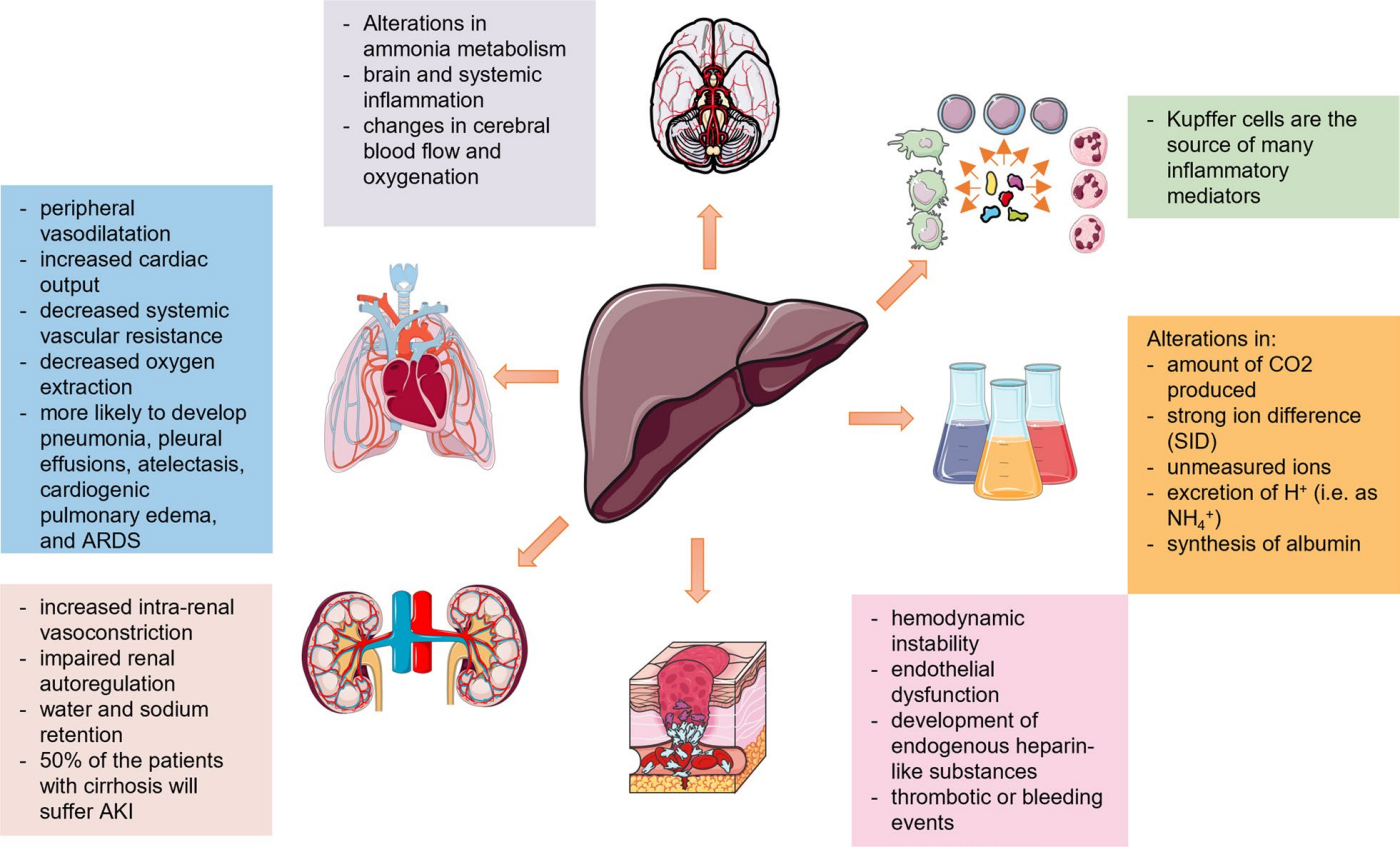
The liver is a “perpetrator” of remote organ damage and development of multiple organ dysfunction syndrome during liver injury

Critically ill patients with liver disease are also more likely to develop pulmonary complications, e.g., acute respiratory distress syndrome (ARDS) [97]. The main mechanisms initiating dysfunction or failure of different organs are further described in [15, 96, 98]. A consensus paper from a North American and European expert panel gives a thorough description of possible organ failures and recommendations for their management [99] (Fig. 1).

The silent period of compensated cirrhosis may turn into a decompensated period (with, e.g., ascites, bleeding from esophageal varices, HE) and is further complicated by cardiopulmonary and renal failure [96]. As described above, the main mechanism triggering renal failure is an alteration in the arterial circulation and volume, which is combined with increased intrarenal vasoconstriction and impaired renal autoregulation. Factors affecting the circulatory status, such as bacterial infections and gastrointestinal bleeding, can reduce renal perfusion and precipitate HRS-AKI [100]. In the case of cardiorespiratory failure, the occurring circulatory changes (increased cardiac output, peripheral vasodilatation, decreased systemic vascular resistance and decreased oxygen extraction) are associated with hypovolemia and impaired tissue perfusion, together with water and sodium retention [100].

The majority of the macrophage population of the body is represented by Kupffer cells in the liver [14]. They act as the first defensive barrier against gut-derived bacteria [101]. The activation of Kupffer cells triggers the release of proinflammatory mediators [102], including TNF-α, IL-1, IL-6 and IL-12. TNF-α and IL-1 can act synergistically to activate the cytokine network, the coagulation cascade, fibrinolysis and neutrophils [103]. IL-6 triggers the synthesis of acute-phase proteins, the production of immunoglobulins, the proliferation and differentiation of T cells, enhanced activity of natural killer cells and the maturation of megakaryocytes [103]. Finally, IL-12 induces the production of interferon-γ in T cells and natural killer cells [14]. These processes can trigger cholestasis [14]. The maximal reduction in bile flow has been reported to occur within the first 24 h after cytokine challenge and can be accompanied by a posttranscriptional mechanism affecting the expression of the hepatobiliary transporters BSEP and MRP2 [104, 105]. In this regard, the development of secondary liver injury, i.e., cholestasis or hypoxic liver injury, is common during sepsis, microcirculatory impairment or drug exposure [106].

Other systems, such as the hemostatic balance in patients with liver injury, might be altered by hemodynamic instability, endothelial dysfunction, the development of endogenous heparin-like substances, leading to either thrombotic or bleeding events [99, 107, 108].

Neurological dysfunction is mainly caused by alterations in ammonia metabolism, brain and systemic inflammation and changes in cerebral blood flow and oxygenation, due to hepatic encephalopathy and/or concomitant infections and electrolyte abnormalities [90, 109]. This might include cerebral edema and intracranial hypertension [97].

Finally, the liver is also responsible for substrate oxidation, metabolism of organic acids (e.g., lactate, ketones, amino acids), metabolism of ammonium and production of plasma proteins [110]. Any alteration of these functions may change the acid–base balance by modifying the amount of CO_2_ produced, the strong ion difference (SID), the concentration of unmeasured ions, the excretion of H^+^ (i.e., as NH_4_^+^) or the synthesis of albumin [111–115]. A retrospective study of 23,795 patients showed that liver “dysfunction” (i.e., SOFA score for liver 1 or 2) was already present on admission in at least 20% of the cases. In the same study, 80% of the non-survivors had an increase in at least one individual organ SOFA score in the four days prior to death [17]. Among patients with organ failure, the highest risk was associated with liver failure (OR 2.587; CI 2.098–3.189).

The association of liver dysfunction and mortality has been suggested by many authors [16, 56, 76, 79–85, 116–119]. Regarding patterns of organ involvement in sepsis, Seymour et al. defined four phenotypes, among which a “hepatic” phenotype with a 28-day mortality rate of 40% among adult patients with sepsis was prognostically the worst [120].

## Conclusions

The definition of liver injury, dysfunction and/or failure is far from a global consensus. Similarly, the cutoff values of prognostic parameters vary. We provided current definitions, epidemiological data and prognostic factors for acute, acute-on-chronic liver failure and secondary liver injury with a focus on the intensive care unit. The reviewed data show an association of liver impairment with extrahepatic organ failure and present liver dysfunction as an underappreciated component for the development of multiple organ failure.

## Data Availability

Not applicable.
